# Genome-Wide Identification and Analysis of the SBP-Box Family Genes under *Phytophthora capsici* Stress in Pepper (*Capsicum annuum* L.)

**DOI:** 10.3389/fpls.2016.00504

**Published:** 2016-04-15

**Authors:** Huai-Xia Zhang, Jing-Hao Jin, Yu-Mei He, Bo-Ya Lu, Da-Wei Li, Wei-Guo Chai, Abid Khan, Zhen-Hui Gong

**Affiliations:** ^1^College of Horticulture, Northwest A&F UniversityYangling, China; ^2^Institute of Vegetables, Hangzhou Academy of Agricultural SciencesHangzhou, China

**Keywords:** *Capsicum annuum* L., SBP-box family genes, Phylogenetic analysis, *Phytophthora capsici*, hormone treatments

## Abstract

SQUAMOSA promoter binding protein (SBP)-box genes encode plant-specific transcription factors that are extensively involved in many physiological and biochemical processes, including growth, development, and signal transduction. However, pepper (*Capsicum annuum* L.) SBP-box family genes have not been well characterized. We investigated SBP-box family genes in the pepper genome and characterized these genes across both compatible and incompatible strain of *Phytophthora capsici*, and also under different hormone treatments. The results indicated that total 15 members were identified and distributed on seven chromosomes of pepper. Phylogenetic analysis showed that SBP-box genes of pepper can be classified into six groups. In addition, duplication analysis within pepper genome, as well as between pepper and *Arabidopsis* genomes demonstrated that there are four pairs of homology of SBP-box genes in the pepper genome and 10 pairs between pepper and *Arabidopsis* genomes. Tissue-specific expression analysis of the *CaSBP* genes demonstrated their diverse spatiotemporal expression patterns. The expression profiles were similarly analyzed following exposure to *P. capsici* inoculation and hormone treatments. It was shown that nine of the *CaSBP* genes (*CaSBP01, 02, 03, 04, 05, 06, 11, 12, and 13*) exhibited a dramatic up-regulation after compatible HX-9 strain (*P. capsici*) inoculation, while *CaSBP09* and *CaSBP15* were down-regulated. In case of PC strain (*P. capsici*) infection six of the *CaSBP* genes (*CaSBP02, 05, 06, 11, 12, and 13*) were arose while *CaSBP14* was down regulated. Furthermore, Salicylic acid, Methyl jasmonate and their biosynthesis inhibitors treatment indicated that some of the *CaSBP* genes are potentially involved in these hormone regulation pathways. This genome-wide identification, as well as characterization of evolutionary relationships and expression profiles of the pepper *CaSBP* genes, will help to improve pepper stress tolerance in the future.

## Introduction

Transcription factors (TFs) are DNA-binding proteins that regulate gene expression at the level of mRNA transcription. They are capable of activating or repressing the transcription of multiple target genes (Yang et al., 2008). In plants, TFs play essential roles in the regulation of many developmental processes (Li et al., 2013). SQUAMOSA promoter binding protein (SBP)-box genes encode a TFs that contain a highly conserved DNA-binding domain termed the SBP domain (Klein et al., 1996; Cardon et al., 1999). This domain comprises approximately 76 amino acid residues that are involved in both DNA binding and nuclear localization, including two zinc-binding sites (Yamasaki et al., 2004). The *AmSBP1* and *AmSBP2* genes of *Antirrhinum majus* were the first SBP-box genes to be discovered based on their ability to interact with the promoter sequence of the floral meristem identity gene SQUAMOSA (Klein et al., 1996). Additional SBP-box genes were later identified, isolated, and characterized in many plants, including *Arabidopsis thaliana* (Cardon et al., 1999), silver birch (Lannenpaa et al., 2004), *Salvia miltiorrhiza* (Zhang et al., 2014), rice (Xie et al., 2006), maize (Chuck et al., 2010), tomato (Salinas et al., 2012), grape (Hou et al., 2013b), and *Gossypium hirsutum* (Zhang et al., 2015).

SQUAMOSA promoter binding protein genes have been found to play a role in the gene regulatory network of the flower formation pathway, and many studies have revealed that these genes are closely related to flower development (Klein et al., 1996; Cardon et al., 1997; Shikata et al., 2009). Moreover, recent studies showed that SBP-box genes are involved in signal transduction and responses to abiotic and biotic stress in many species. For instance, *AtSPL14* has been found to be involved in determining sensitivity to the programmed cell death-inducing fungal toxin fumonisin B1 (Stone et al., 2005). *AtSPL2* (At5g43270), which is modified in transgenic *Arabidopsis* overexpressing the JASMONATE CARBOXYL METHYLTRANSFERASE gene (AtJMT) response to jasmonic acid mediated resistance pathway (Jung et al., 2007). *VpSBP5* likely participates in regulating resistance to *Erysiphe necator* by activating the SA-induced systemic acquired resistance pathway and MeJA-induced wound signaling pathway in grapes (Hou et al., 2013b). However, little is currently known about the SBP-box genes in pepper, especially regarding resistance to *Phytophthora* blight.

Pepper (*Capsicum annuum* L.) is one of the most important vegetable crops worldwide. The *Phytophthora* blight in pepper is caused by the oomycete *Phytophthora capsici*, which mainly attacks the roots and is one of the most destructive diseases worldwide (Hausbeck and Lamour, 2004; Zhang et al., 2013), as it also infects tomato, eggplant, cucumber, watermelon, pumpkin, squash, cocoa, and other plants (Biles et al., 1995; Oelke and Bosland, 2003). The pathogen can affect the plant at any stage of development causing damping-off, seedling blight, and wilting, followed by plant death. Infected plants have rapidly expanding water-soaked lesions (Kousik et al., 2012). Analysis of *C. annuum* SBP-box (*CaSBP*) genes in response to *P. capsici* and hormones is therefore important for identification of candidate genes in pepper.

In the current study, we report the genome-wide identification and characterization of SBP-box genes in the pepper genome, including sequence alignment, phylogenetic analysis, intron-exon structure, chromosomal location, and synteny. Moreover, we investigated the expression patterns of *CaSBP* genes in various pepper tissues/organs, as well as the transcriptional responses of *CaSBP* genes in the roots of different *P. capsici*. Five *CaSBP* genes were selected based on their expression patterns after inoculation with *P. capsici*, and their expression profiles were assessed following treatment with different plant hormones and corresponding biosynthetic inhibitors. Our findings lay the foundation for future research into the functions of disease-related genes from the SBP-box gene family in pepper.

## Materials and Methods

### Identification and Annotation of SBP-Box Genes in Pepper

A hidden Markov model (HMM) profile of the SBP domain (Accession no. PF03110) was downloaded from the Pfam database^1^ This domain was used to query the CM334 (*C. annuum*) Genome Database and Zunla-1 (*C. annuum*) Genome Database^2^ (V1.55) with the BLASTP program. All hits with an *E*-value < 1.5e-7 were identified. All non-redundant protein sequences were searched for the SBP domain using NCBI’s conserved domain database^3^ Candidate *CaSBP* genes were aligned with DNAMAN software (Version 5.0), and genes with differing sequences between the two cultivars were identified (Guo et al., 2015). Primers (**Supplementary Table S1**) were designed to amplify the sequences with Primer Premier 5.0 (Premier Biosoft International, Palo Alto, CA, USA), and CM334 and Zunla-1 sequences for the same gene were then aligned to confirm the correct sequences. In order to compute the theoretical isoelectric point (pI) and protein molecular weight (MW), the deduced amino acid sequences were analyzed using DNAStar Lasergene software (Version 7.1). Names of putative *CaSBP* genes were assigned based on chromosomal order.

### Sequence Alignments, Phylogenetic Analysis, and Intron/Exon Structure Determination

Multiple amino acid sequence alignment was performed using DNAMAN software (Version 5.0). The sequence logo was obtained using the online platform Weblogo^4^ for conserved sequences. Phylogenetic trees were constructed using MEGA 6.0 with the maximum likelihood method and 1000 bootstrap replicates. Intron/exon structures were determined by aligning coding sequences to their corresponding genomic sequences. A diagram of intron/exon structures was obtained using the method described by Guo et al. (2015), which depicts both exon positions and gene lengths.

### Chromosomal Location and Duplication Analysis

Chromosomal location information was derived from the pepper genome^5^, and genes were mapped to chromosomes using MapDraw (Liu and Meng, 2003) and their physical chromosome positions. Identification of duplicate genes within the pepper genome and between pepper and *Arabidopsis* was performed using the following criteria described by Gu et al. (2002): (1) the FASTA-alignable region between the two proteins had to be greater than 80% of the longer protein, and (2) the identity (I) between the two proteins had to be ≥30% if the alignable region was longer than 150 aa and ≥0.01n + 4.8 L^-0.32(1+exp(-L/1000)^ (Rost, 1999) if otherwise, where *n* = 6 and L is the alignable length between the two proteins (Rost, 1999; Gu et al., 2002).

### Plant Materials and Seedling Treatment

In this study, we used the pepper cultivar AA3 (provided by the pepper research group, College of Horticulture, Northwest A&F University, Yangling, China), which is susceptible to a compatible HX-9 strain and resistant to an incompatible PC strain of *P. capsici*. Plants were grown in a growth chamber at 22/18°C day/night temperature and 16/8 h day/night photoperiod. Various vegetative and reproductive tissues, including roots, stems, leaves, flowers, green fruits, and mature fruits were collected and stored at -80°C for tissue-specific experiments.

Pepper plants at the 8–10 true leaves stage were inoculated with compatible and incompatible strains of *P. capsici* using the root-drenching method, as described by Wang et al. (2013a), while control plants were inoculated with sterile distilled water. Root samples were taken at 0, 6, 12, 24, and 48 h and stored at -80°C. Seedlings were treated with 100 μM SA synthesis inhibitor (paclobutrazol, PBZ; Liu et al., 2006) or 50 μM MeJA synthesis inhibitor (salicylhydroxamic acid, SHAM; Dong et al., 2009). After 24 h of treatment, plants were treated with the corresponding inducer, 5 mM SA or 50 μM MeJA, using the method described by Yin et al. (2014). A mixture of 0.5% Tween and 0.1% alcohol was used as a control for PBZ and SHAM treatment, while PBZ and SHAM treatment alone (no inducer) was also used as an induction control. Leaves were harvested at 0, 3, 6, 9, 12, 24, and 48 h and were quickly frozen with liquid nitrogen and stored at -80°C.

### RNA Extraction and Quantitative Real-Time PCR

Total RNA was isolated using the method described by Guo et al. (2012), and cDNA was synthesized according to the manufacturer’s instructions of PrimeScript Kit (Takara, Dalian, China). The cDNA was then diluted to 50 ng/μL with ddH_2_O. For quantitative real-time PCR (qRT-PCR), primer pairs (**Supplementary Table S2**) for *CaSBP* genes were designed by Primer Premier 5.0, and their specificities was assessed using NCBI Primer BLAST^6^ The ubiquitin binding-protein gene (*UBI-3*) from pepper was used as reference (Schmittgen and Livak, 2008). qRT-PCR was performed as described by Guo et al. (2015) on the iQ5.0 Bio-Rad iCycler thermocycler (Bio-Rad, Hercules, CA, USA) using SYBR Green Supermix (Takara, Dalian, China). qRT-PCR cycling conditions were as follows: pre-denaturation at 95°C for 1 min, followed by 40 cycles of denaturation at 95°C for 10 s, annealing at 56°C for 30 s, and extension at 72°C for 30 s. The fluorescent signal was measured at the end of each cycle, and melting curve analysis was performed by heating the PCR product from 56 to 95°C in order to verify the specificities of the primers. Three independent biological replicates were carried out. The relative expression levels of pepper *SBP* genes were calculated using the -ΔΔCT method (Schmittgen and Livak, 2008).

## Results

### Genome-Wide Identification and Annotation of SBP-Box Genes in Pepper

The identification of SBP-box gene family members in pepper was performed in three steps. In the first step, the HMM profile of the SBP domain was used as a BLAST query against the pepper genome. A total of 15 and 16 candidate SBP-box genes were obtained from pepper cultivars CM334 and Zunla-1, respectively. In the second step, CM334 and Zunla-1 genes were compared, and sequences were re-amplified to verify the corresponding genes. One candidate gene (Gene ID: Capana03g002994) found in Zunla-1 was discarded due to poor identification in comparison with the corresponding sequence in CM334. In the final step, each predicted SBP-box protein sequence was confirmed to have a conserved SBP domain using an NCBI search. As a result, 15 candidate SBP-box genes were confirmed and named based on their chromosomal order in pepper (**Table 1**). The *CaSBP* coding sequences ranged from 336 bp (*CaSBP08*) to 3024 bp (*CaSBP06*), while deduced proteins ranged from 111 to 983 amino acids in length and from 13.11 to 108.67 kDa in MW. The predicted isoelectric points (pI) of the *CaSBP*s varied from 5.61 to 9.54.

**Table 1:** Characterization of SQUAMOSA promoter binding protein (SBP)-box family genes in pepper.

Gene	SGN locus	Chr.	Introns	AA	WT	PI
CaSBP01	Capana01g002647	1	3	463	50.32	8.84
CaSBP02	Capana01g002832	1	11	930	103.34	5.61
*CaSBP03*	Capana01g003073	1	9	796	89.15	6.73
*CaSBP04*	Capana01g003445	1	2	290	33.21	9.01
*CaSBP05*	Capana02g001917	2	1	136	15.72	8.27
*CaSBP06*	Capana05g002237	5	10	983	108.67	7.45
*CaSBP07*	Capana07g001731	7	1	183	20.79	9.54
*CaSBP08*	CA07g17550 ▲	7	0	111	13.11	7.72
*CaSBP09*	CA08g03640 ▲	8	0	144	16.32	9.04
*CaSBP10*	Capana10g000507	10	1	141	16.27	7.31
*CaSBP11*	Capana10g000709	10	2	507	55.17	8.81
*CaSBP12*	Capana10g000886	10	2	299	33.71	8.48
*CaSBP13*	Capana10g002379	10	2	367	39.57	8.53
*CaSBP14*	Capana11g002003	11	2	548	60.19	7.41
*CaSBP15*	CA11g04690 ▲	11	0	144	16.18	9.46


*Chr, chromosome location; AA, amino acid; Mol. Wt., molecular weight (kDa); pI, isoelectric point. SGN loci marked with triangle (▲) are from CM334 genome, others are from Zunla-1 genome.*

### Sequence Alignments, Phylogenetic Analysis, and Intron/Exon Structure Determination

**FIGURE 1:**
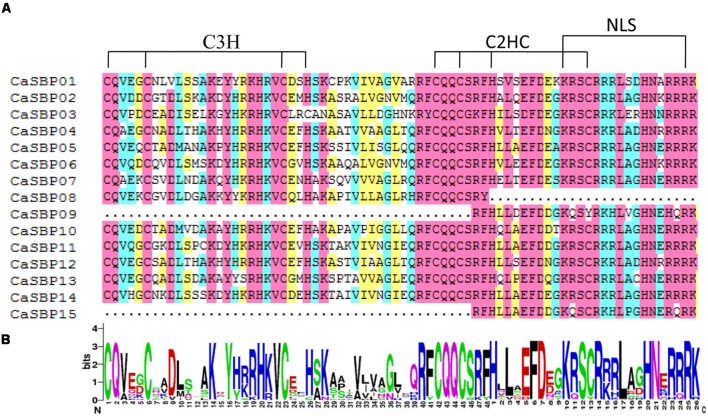
**SBP domain alignment in *CaSBP*s.** **(A)** Multiple alignment of the SQUAMOSA promoter binding protein (SBP) domains of pepper SBP-box proteins obtained with DNAMAN software. The two conserved zinc-finger structures (C3H, C2HC) and NLS are indicated. **(B)** Sequence logo of the SBP domain of *CaSBP*s. The overall height of each stack represents the degree of conservation at each position, while the height of the letters within each stack indicates the relative frequency of the corresponding amino acid.

Multiple sequence alignment of full-length protein sequences was performed to analyze the domain structures of CaSBPs in detail. The SBP domain is the only conserved domain shared by all CaSBPs (**Figure 1A**) and was highly similar across proteins, with high or complete conservation at certain positions (**Figure 1B**). All CaSBPs exhibit two zinc finger-like structures (C3H, C2HC) and a highly conserved bipartite nuclear localization signal (NLS), with the exception of CaSBP08, which lacks the C2HC and NLS. In addition, CaSBP09 and CaSBP15 are also lacking C3H, as the second zinc finger-like structure partially overlaps the NLS, as previously reported (Birkenbihl et al., 2005).

**FIGURE 2:**
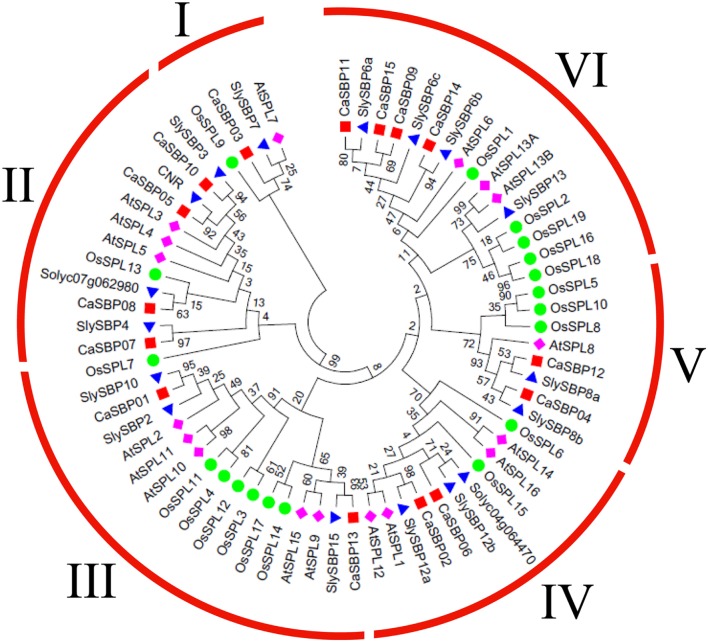
**Phylogenetic analysis of pepper and other plant SBPs.** A phylogenetic tree was constructed with SBP domain protein sequences from pepper, tomato, *Arabidopsis*, and rice. The SBP domain sequences, accession numbers/locus IDs, and data sources of all genes used for phylogenetic tree construction are listed in **Supplementary Table S3**.

**FIGURE 3:**
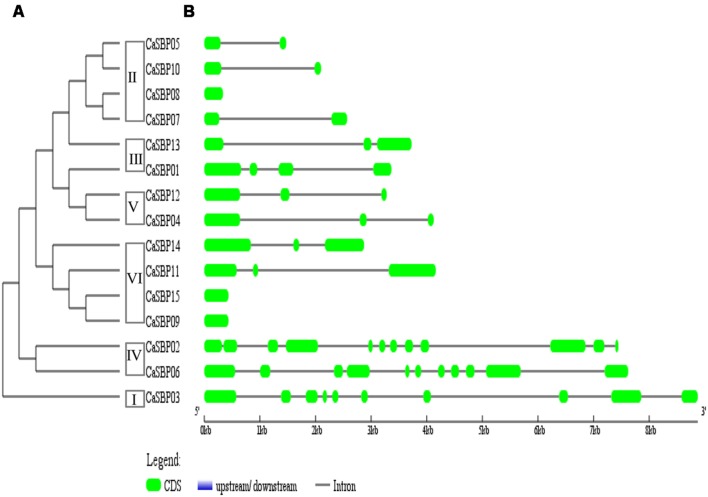
**Phylogenetic analysis and intron/exon structures of *CaSBP* genes.** **(A)** A phylogenetic tree was constructed with pepper SBP domain protein sequences. **(B)** Exons and introns are indicated by green boxes and black horizontal lines, respectively.

To investigate the evolutionary relationship between *CaSBP* genes and SBP-box genes from *Arabidopsis*, tomato (*Solanum lycopersicum*), and rice (*Oryza sativa*), we constructed a phylogenetic tree using the maximum likelihood algorithm (**Figure 2**), with 17 *Arabidopsis* genes, 17 tomato genes, and 19 rice genes (**Supplementary Table S3**). Only the protein sequences of the highly conserved SBP domains were used for phylogenetic analysis, as alignment of the full-length protein sequences revealed that only the SBP domains were conserved (Hou et al., 2013a). According to the unrooted phylogenetic tree, CaSBP proteins clustered with those of the other species into six distinct groups (I–VI; **Figure 2**), with each group containing at least one protein from each species. The plant SBP-box gene family is evolutionarily diversified. An unrooted phylogenetic tree was also constructed using only the SBP domains from CaSBPs (**Figure 3A**).

Intron/exon structures of all 15 *CaSBP* genes were generated based on genome sequences and corresponding coding sequences (**Figure 3B**). Intron/exon structure diagrams revealed high variation in the number of introns, from zero (*CaSBP08, CaSBP09*, and *CaSBP15*) to 11 (*CaSBP02*). Based on the CaSBP tree (**Figure 3A**), class I proteins contain nine introns, class II contains 0–1, class III contains 2–3, class IV contains 10–11, class V contains 2, and class VI contains 0–2 introns.

### Chromosomal Location and Duplication Analysis

**FIGURE 4:**
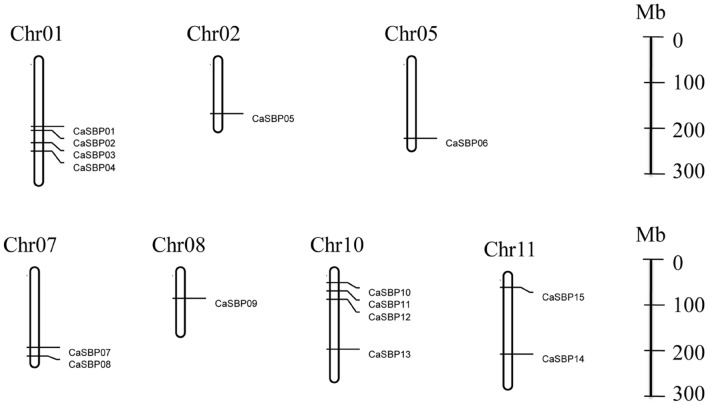
**Chromosome mapping of *SBP* genes in pepper.** Chromosome numbers are indicated at the top of each bar. Scale is represented in megabases (Mb).

We found that *CaSBP* genes were located on seven of the twelve pepper chromosomes (**Figure 4**): chromosomes 1, 2, 5, 7, 8, 10, and 11 (**Table 1**). Chromosomes 1 and 10 contained the most *CaSBP* genes, with four genes each (*CaSBP01*–*CaSBP04* and *CaSBP10*–*CaSBP13*, respectively), followed by chromosomes 7 and 11, with two genes each (*CaSBP07*–*CaSBP08* and *CaSBP14*–*CaSBP15*, respectively).

**FIGURE 5:**
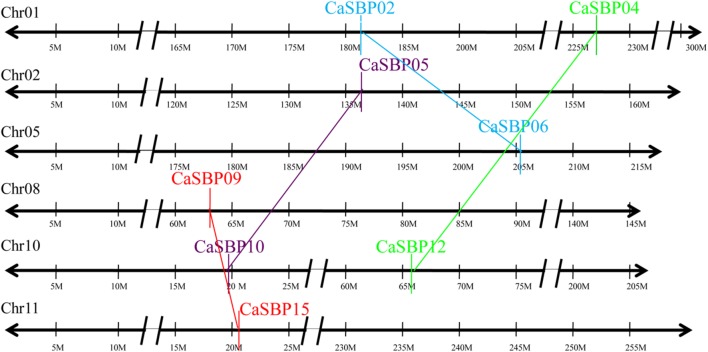
**Duplication analysis of pepper SBP-box genes.** The positions of duplicated *CaSBP* genes are depicted on pepper chromosomes 1, 2, 5, 8, 10, and 11. Colored lines connecting two chromosomal regions indicate duplicated regions between pepper chromosomes.

**FIGURE 6:**
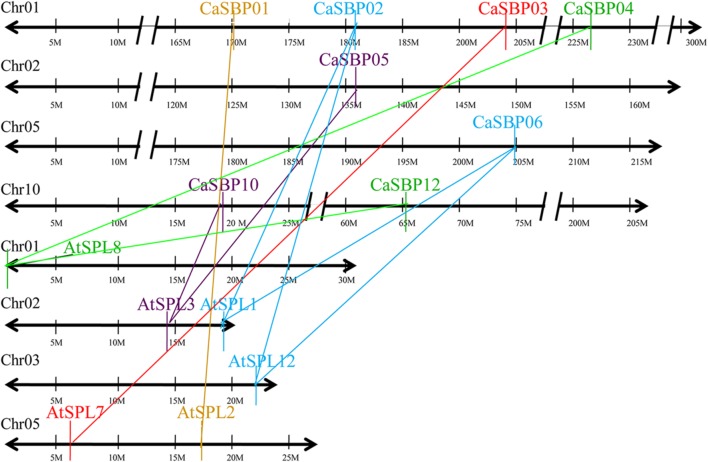
**Duplication analysis of SBP-box genes between pepper and *Arabidopsis* genomes.** The positions of related *CaSBP* and *AtSPL g*enes are depicted on pepper chromosomes 1, 2, 5, and 10 and *Arabidopsis* chromosomes 1, 2, 3, and 5. Colored lines connecting two chromosomal regions indicate duplicated regions between pepper and *Arabidopsis* chromosomes.

Duplication analysis, using the criteria described by Gu et al. (2002), confirmed that four pairs of pepper SBP-box genes (*CaSBP02*/*06*, *CaSBP04*/*12*, *CaSBP05*/*10*, and *CaSBP09*/*15*) were the result of interchromosomal segmental duplications (**Figure 5**). Because *Arabidopsis* is a popular model plant and the functions of several *Arabidopsis* SBP-box genes have been well characterized, we also used the same criteria to identify SBP-box gene orthologs between the pepper and *Arabidopsis* genomes to further study the origin, evolutionary history, and putative function of the pepper SBP-box genes. Based on this analysis, we identified ten pairs of CaSBP–AtSPL orthologs (*CaSBP01*–*AtSPL2*, *CaSBP02–AtSPL1*/*12*, *CaSBP03–AtSPL7*, *CaSBP04*/*12–AtSPL8*, *CaSBP05*/*10–AtSPL3*, and *CaSBP06–AtSPL1/12*) (**Figure 6**), indicating that many of pepper SBP-box genes and their *Arabidopsis* counterparts appear to be derived from a common ancestor. According to these results, we were able to infer the functions of several pepper SBP-box genes based on their *Arabidopsis* homologs, facilitating research into the roles of SBP-box genes in pepper.

### Expression Profiles of *CaSBP* Genes in Pepper Tissues

**FIGURE 7:**
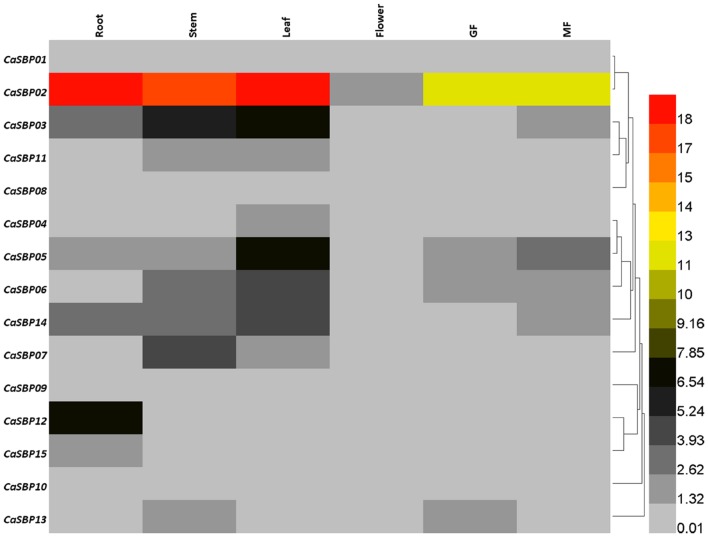
**Tissue-specific expression analysis of pepper SBP-box genes.** Analyzed tissues included root, stem, leaf, flower, green fruit (GF), and mature fruit (MF).

In order to provide additional information on the functions of SBP-box genes in pepper, we investigated their expression profiles in various organs and at different stages of fruit development in cultivar AA3 via qRT-PCR with transcript-specific primers (**Supplementary Table S2**). Generally, the expression patterns of *CaSBP* genes can be classified into two types (**Figure 7**). The minority of *CaSBP* genes, specifically *CaSBP01*, *CaSBP08*, *CaSBP09*, and *CaSBP10*, exhibited low-level, constitutive expression in all pepper tissues/organs examined. The remaining *CaSBP* genes were only expressed in certain tissues or organs. *CaSBP02* was the most highly expressed SBP-box gene in the examined tissues. In general, the expression of *CaSBP* genes was highest in the leaf, followed by the stem, root, green fruit, mature fruit, and flowers.

### Expression Analysis of *CaSBP* Genes under *P. capsici* and Hormone Treatments

**FIGURE 8:**
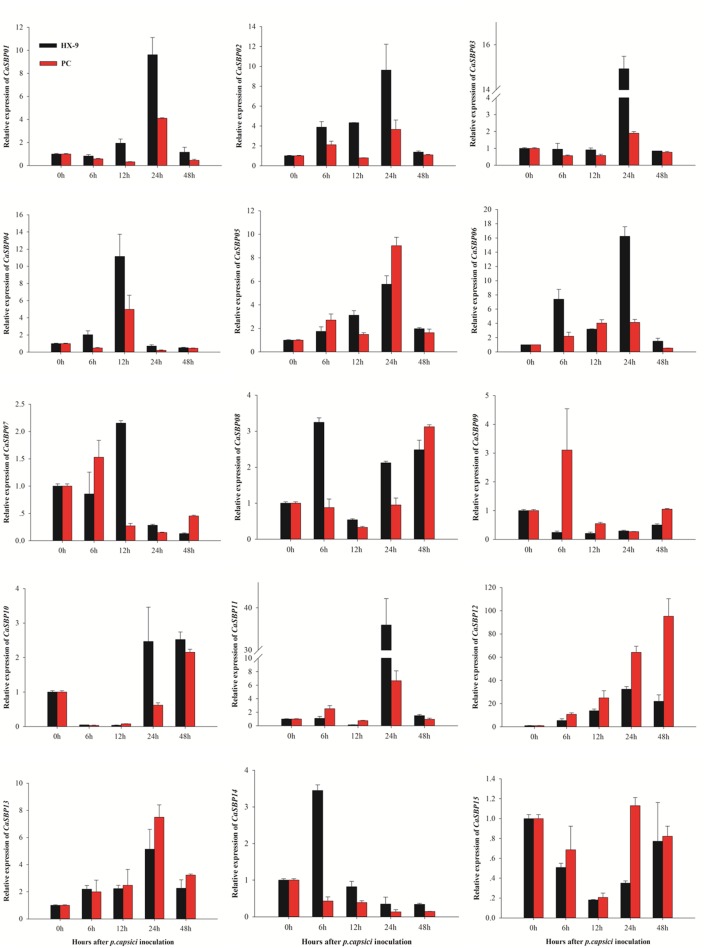
**Expression profiles of *CaSBP*s in response to inoculation with compatible or incompatible *Phytophthora capsici* strains.** Mean values and SDs for three replicates are shown.

To investigate the effect of *P. capsici* infection on the expression of *CaSBP* genes, roots from the AA3 cultivar were inoculated with compatible and incompatible *P. capsici* strains, and changes in gene expression were analyzed using qRT-PCR (**Figure 8**). The results indicate that after inoculation with either the compatible or incompatible strain, four *CaSBP* genes (*CaSBP02*, *CaSBP05*, *CaSBP06*, and *CaSBP13*) were up-regulated 0–24 h post-inoculation and subsequently down-regulated, while *CaSBP04* was up-regulated 0–12 h and then down-regulated. Similarly, *CaSBP14* was up-regulated 0–6 h post-inoculation and subsequently down-regulated. Following inoculation with just the incompatible strain, four genes (*CaSBP01, CaSBP03, CaSBP05*, and *CaSBP08)* exhibited down-regulation 0–12 h post-inoculation, followed by up-regulation to 24 h and subsequent down-regulation again. *CaSBP10* and *CaSBP11* exhibited the same pattern but following inoculation with the compatible strain only. Following compatible strain inoculation, four genes (*CaSBP01, CaSBP02, CaSBP03*, and *CaSBP12*) were up-regulated 0–24 h and subsequently down-regulated, while two genes (*CaSBP07* and *CaSBP09*) were up-regulated 0–6 h after inoculation with the incompatible strain and then down-regulated. Moreover, *CaSBP09* exhibited consistent down-regulation following inoculation with the compatible strain, and *CaSBP12* exhibited up-regulation 0–48 h after inoculation with the incompatible strain. Generally, the expression patterns of *CaSBPs* after inoculation with *P. capsici* can be divided into five categories. The first and second categories contain one gene each, *CaSBP04* and *CaSBP10*, whose expression peaked at 12 and 48 h, respectively, after inoculation with either the compatible or incompatible strain. The third category contains seven genes (*CaSBP01–CaSBP03*, *CaSBP05*, *CaSBP06*, *CaSBP11*, and *CaSBP13*) whose expressions peaked 24 h after inoculation with either the compatible or incompatible strain. The fourth category contains two genes, *CaSBP08* and *CaSBP12*, whose expressions peaked earlier following inoculation with the compatible strain than following inoculation with the incompatible strain. The fifth category contains four genes (*CaSBP07*, *CaSBP09*, *CaSBP14*, and *CaSBP15*), whose expressions were down-regulated 12 h after inoculation with either the compatible or incompatible strain.

**FIGURE 9:**
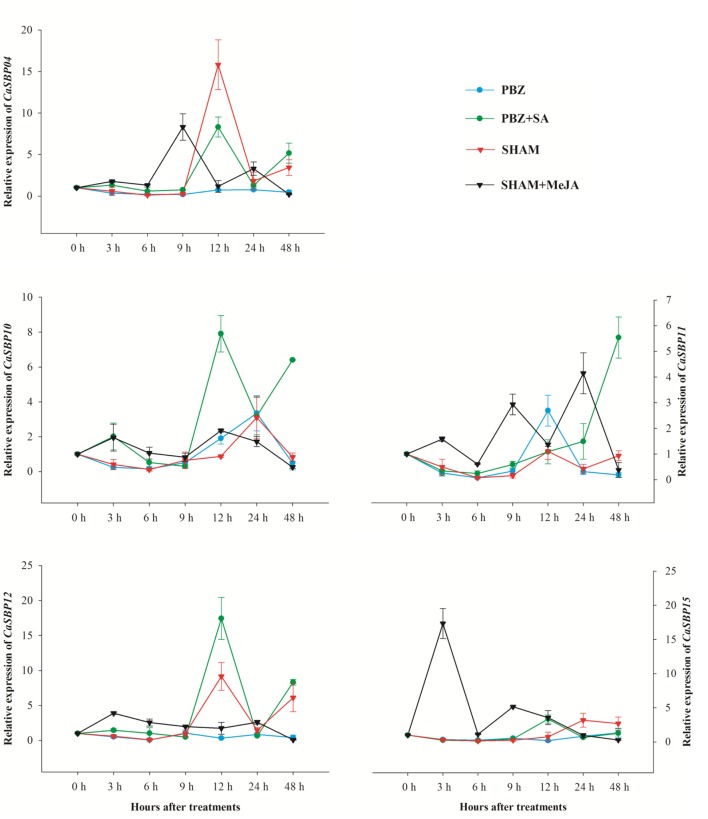
**Expression profiles of *CaSBP*s in response to treatment with SA or MeJA hormones and the corresponding inhibitors PBZ or SHAM.** Mean values and SDs for three replicates are shown.

To investigate the expression patterns of *CaSBPs* in response to treatment with various signal molecules, five representative genes (*CaSBP04*, *CaSBP10*–*12*, and *CaSBP15*), one from each of the five categories above, were treated with SA inhibitor (PBZ) or MeJA inhibitor (SHAM), and changes in gene expression were analyzed using qRT-PCR (**Figure 9**). Results showed that the expression of all five genes was rapidly down-regulated 0–6 h after treatment with SA inhibitor (PBZ) or MeJA inhibitor (SHAM), reaching the lowest level at 6 h. After 24 h of treatment, the corresponding inducer (SA or MeJA) was applied. Subsequently, the expression levels of the five genes after SA treatment peaked at 12 h, with the exception of *CaSBP11*, which peaked at 48 h. Following MeJA treatment, expression levels of the five genes peaked earlier than 12 h.

## Discussion

Most evidence suggests that SBP-box genes play central roles in plant development, signal transduction, and defense processes (Schwarz et al., 2008; Shikata et al., 2009; Hou et al., 2013b). Benefitting from the availability of genome sequences, the functions of SBP-box genes have been characterized in many plants, including *Arabidopsis*, *S. miltiorrhiza* (Zhang et al., 2014), rice (Yang et al., 2008), tomato (Yang et al., 2008), *Populus trichocarpa* (Li and Lu, 2014), grape (Hou et al., 2013a), apple (Li et al., 2013), *G. hirsutum* (Zhang et al., 2015), *Prunus mume* (Xu et al., 2015), castor bean (Zhang and Ling, 2014), and citrus (Shalom et al., 2015). However, the functions of pepper SBP-box TFs are still unknown. In this study, through genome-wide identification and molecular cloning, we discovered the first set of *CaSBP* genes (**Table 1**). In total, we identified 15 *CaSBP*s in pepper, a number similar to that found in *S. miltiorrhiza* (Zhang et al., 2014), *P. mume* (Xu et al., 2015), castor bean (Zhang and Ling, 2014), and citrus (Shalom et al., 2015).

Phylogenetic tree analysis showed that *SBP*s from representative plants are clustered into six groups, with *CaSBP* genes distributed across all six groups (**Figure 2**). In addition, each group contains at least one gene from *Arabidopsis*, tomato, and rice. *CaSBP* genes are more closely related to genes from tomato or *Arabidopsis* than to rice SBP-box genes, reflecting the fact that *Arabidopsis*, tomato, and pepper are eudicots and diverged more recently from a common ancestor (Li et al., 2013). These results indicate that although plant SBP-box genes may be derived from a common ancestor, many have undergone distinct patterns of differentiation with the divergence of different lineages. Gene structure analyses showed that within the same phylogenetic group, most *CaSBP* genes shared similar intron/exon structures, indicating that the evolution of SBP domains may be closely related to the diversification of gene structures, as described previously in tomato (Wan et al., 2013), rice (Xie et al., 2006), apple (Li et al., 2013), and grape (Hou et al., 2013a). *CaSBP* genes are distributed across seven of the twelve pepper chromosomes, with no *CaSBP* genes on chromosomes 3, 4, 6, 9, or 12. Similarly, only chromosomes 6, 8, 9, and 11 lack *SBP* genes in tomato, suggesting that *SBP* genes may have been widely distributed across the genome of the *Solanaceae* common ancestor.

Gene duplication events include tandem, segmental, and whole-genome duplications, and they have played crucial roles in the evolution of various organisms (Xu et al., 2012). In the SBP-box gene family, there are two pairs of *Arabidopsis* (*AtSPL1*/*12* and *AtSPL4/5*), six pairs of rice genes (*OsSPL1/6*, *OsSPL3/12*, *OsSPL4/11*, *OsSPL5/10*, *OsSPL14/17*, and *OsSPL16/18*), eight pairs of apple genes (*MdSBP1B/9*, *MdSBP4A-B/20*, *MdSBP8/27A-B*, *MdSBP10/21*, *MdSBP10/22*, *MdSBP11/21*, *MdSBP12/23*, and *MdSBP13/15*), and six pairs of grape genes (*VvSBP2/15*, *VvSBP3/12*, *VvSBP5/7*, *VvSBP9/11, VvSBP9/18*, and *VvSBP11*/*18*) located within segmental duplications (Xie et al., 2006; Li et al., 2013; Hou et al., 2013a). Similarly, we used the criteria described by Gu et al. (2002) and confirmed that four pairs of pepper SBP-box genes (*CaSBP02/06*, *CaSBP04/12*, *CaSBP05/10*, and *CaSBP09/15*) are located in putative segmental duplications. Therefore, it is clear that segmental duplications have played an important role in the expansion of the plan SBP-box gene family.

Comparative genomic analysis is a relatively rapid and effective way to transfer genomic knowledge acquired in one taxon to another, whose genome structure, function, and/or evolution are less known (Lyons et al., 2008). Thus, putative functions of pepper SBP-box genes can be inferred via comparison with orthologs in well-studied model plants such as *Arabidopsis*. In this study, duplication analysis between pepper and *Arabidopsis* indicated that ten pairs of SBP-box genes (*CaSBP01*/*AtSPL02*, *CaSBP02-06*/*AtSPL1-12*, *CaSBP03/AtSPL7*, *CaSBP04-12*/*AtSPL8*, and *CaSBP05-10/AtSPL3*) are located in syntenic genomic regions and represent putative orthologs (**Figure 6**). To date, the majority of *Arabidopsis* SBP-box genes, including *AtSPL2* (Shikata et al., 2009), *AtSPL3* (Yamaguchi et al., 2009), *AtSPL4* (Jung et al., 2011), *AtSPL5* (Jung et al., 2011), *AtSPL6* (Padmanabhan et al., 2013), *AtSPL7* (Yamasaki et al., 2009), *AtSPL8* (Zhang et al., 2007; Xing et al., 2010), *AtSPL9* (Cui et al., 2014), *AtSPL10* (Shikata et al., 2009), *AtSPL11* (Shikata et al., 2009), *AtSPL13* (Martin et al., 2010), *AtSPL14* (Stone et al., 2005), and *AtSPL15* (Schwarz et al., 2008) have been functionally characterized. Therefore, the functions of several *CaSBP* gene homologs, such as *CaSBP01*–*CaSBP05*, *CaSBP10*, and *CaSBP12*, can be predicted based on their *Arabidopsis* counterparts. Further experiments are necessary to confirm these functions.

In order to further reveal the possible roles of *CaSBP* genes in pepper growth and development, the expression profile of each *CaSBP* gene was investigated in six different tissues. Results indicate that *CaSBP* genes exhibit different expression patterns (**Figure 7**). While a few *CaSBP* genes (*CaSBP01*, *CaSBP08*–*CaSBP10*) demonstrated low-level, constitutive expression in all tissues or organs examined, the majority were limited to certain tissues/organs, with *CaSBP02* exhibiting the highest expression across all tissues. The transcription levels of *CaSBP03*, *CaSBP05*, and *CaSBP06* were also higher than other *CaSBP* genes in root, stem, and leaf, consistent with the results of previous sequencing in hot peppers (Kim et al., 2014). In addition, the expression of *CaSBP* genes in flowers and fruits was lower than that in roots, stems, and leaves, similar to results from grapes (Hou et al., 2013a), which may indicate that *CaSBP* genes play a role in the transition from vegetative to reproductive growth. Unlike *MdSBP* genes in apple (Li et al., 2013), however, *CaSBP* expression patterns were not correlated with gene location, gene length, gene structure, or gene sequence.

Most *CaSBP* genes were up-regulated after inoculation with compatible and incompatible *P. capsici*. Specifically, *CaSBP02*, *CaSBP05*, *CaSBP06*, *CaSBP11*, *CaSBP12*, and *CaSBP13* exhibited significantly higher expression under *P. capsici* stress conditions in pepper roots (**Figure 8**). In addition, the transcript levels of *CaSBP05*, *CaSBP12*, and *CaSBP13* were up-regulated more rapidly and more intensely following inoculation with the strain than with the compatible strain. Recent studies have indicated that a novel peroxidase (*CanPOD*) and oxysterol-binding protein (*CanOBP*) genes, which are involved in the defense response to *P. capsici* infection, exhibit expression patterns similar to these *CaSBP*s (Liu, 2009; Wang et al., 2013b). Moreover, similar expression patterns are also found in some defense-related genes – such as the disease-associated protein gene (*CABPR1*), β-1,3-glucanase gene (*CABGLU*), and peroxidase gene *(CAPO1*) – in pepper roots after inoculation with compatible and incompatible *P. capsici* (Wang, 2013). However, according to Kim and Hwang (2000), the expression of *CABPR1* is higher in the compatible interaction than in the incompatible interaction. While differences in expression changes between *CaSBP* and *CABPR1* genes may be due to differences in inoculation of the *P. capsici* strains or to differences in the compatibility systems, it suggests that these genes are related to the pepper’s resistance to *P. capsici*. Phylogenetic tree analysis showed that *CaSBP02* and *CaSBP06* exhibited a close relationship with *AtSPL14*, which has been found to be involved in programmed cell death and plays a role in sensitivity to fumonisin B1 (Stone et al., 2005). Moreover, the ortholog of *AtSPL14* and *VpSBP5* is likely to participate in regulating resistance to *E. necator* (Hou et al., 2013a). It also has been reported that *AtSPL* genes are co-expressed with two TFs, *TGA1*, and *WRKY65*, which are induced by pathogens and regulate the expression of several stress-responsive genes, such as pathogenesis-related 1 protein (*PR-1*) and GLUTATHIONE S-TRANSFERASE 6 (*GST6*; Wang et al., 2009). Based on the above results, we speculate that these *SBP* genes may be involved in disease resistance, but this will need to be verified.

The signal transduction pathway mediated by salicylic acid (SA) and methyl jasmonate (MeJA) is linked to the plant defense response (Thomma et al., 2001; An et al., 2008; Choi and Hwang, 2011). SA typically mediates basal defense to biotrophic pathogens (Thomma et al., 2001), while MeJA generally controls defensive reactions to necrotrophs (Glazebrook, 2005). Therefore, we investigated the responses of five representative *CaSBP*s (*CaSBP04*, *CaSBP10*, *CaSBP11*, *CaSBP12*, and *CaSBP15*) to plant hormone signals by examining their transcript levels in pepper leaves upon treatment with SA or MeJA and their corresponding biosynthesis inhibitors. The expression levels of most genes peaked at 12 h following SA treatment, the exception being *CaSBP11*, which peaked at 48 h. Following MeJA treatment, the maximum expression of all five genes occurred earlier than after SA treatment. It has been reported that SA and MeJA can induce the expression of defense-related gene *PR-1* in tobacco (Xu et al., 1994; Vidal et al., 1997). Moreover, SA induces the recruitment of *trans*-activating TGA factors to the promoter of a defense gene in *Arabidopsis* (Johnson et al., 2003). The *Arabidopsis* SBP-box gene *AtSPL2* and the grape SBP-box gene *VpSBP5* also exhibit responsiveness to biotic stress signaling hormones (Jung et al., 2007; Hou et al., 2013b). Therefore, we speculate that these genes may be involved in the response to various plant stress hormones, particularly the MeJA-induced necrotroph pathway.

## Conclusion

In this study, we identified SBP-box genes in pepper and analyzed them via sequence alignment, phylogenetic analysis, intron/exon structure, chromosomal location, and duplication analysis. We also assessed the expression profiles of pepper *SBP* genes across different tissues (root, stem, leaf, flower, and fruit) and under infection with both compatible and incompatible *P. capsici* strains and hormone treatment. Most *CaSBP* genes are expressed at low levels under normal circumstances and are induced by *P. capsici* and hormones, indicating that these genes may be involved in the resistance pathways mediated by *P. capsici*, SA, and MeJA. Candidate pepper SBP-box genes from this analysis should be further functionally characterized for deeper understanding of the precise regulatory checkpoints that operate during stress responses.

## Author Contributions

H-XZ, W-GC, and Z-HG conceived and designed the experiments. H-XZ, J-HJ, Y-MH, D-WL, B-YL, and AK performed the experiments. H-XZ analyzed the data. W-GC and Z-HG contributed reagents/materials/analysis tools. H-XZ wrote the paper.

## Conflict of Interest Statement

The authors declare that the research was conducted in the absence of any commercial or financial relationships that could be construed as a potential conflict of interest.

## Footnotes

1 http://www.sanger.ac.uk.

2 http://peppergenome.snu.ac.kr/blast.php

3 http://www.ncbi.nlm.nih.gov/Structure/cdd/wrpsb.cgi.

4 http://weblogo.berkeley.edu

5 http://peppergenome.snu.ac.kr/

6 http://www.ncbi.nlm.nih.gov/tools/primer-blast/index.cgi?LINK_LOC=BlastHome.
