# Supplementary material for: Genome-Wide Identification and Analysis of the SBP-Box Family Genes under Phytophthora capsici Stress in Pepper (Capsicum annuum L.)
Source: Front Plant Sci. 2016 Apr 15;7:504. doi: 10.3389/fpls.2016.00504 (PMC4832253; doi:10.3389/fpls.2016.00504)
Supplement: Supplementary file 1 [file Table_1.DOC]

**Table S1** Primers names and their sequences used in study for confirm the correct sequences

| Oligo Name | Primer Abbreviation | Primer Sequence (5^’-^3’) |
| --- | --- | --- |
| CaSBP02 | SBPzunla-2-F  SBPzunla-2-R1  SBPzunla-2-F2  SBPcm334-2-R | ATGGAAGCGAGTGTTGGAGAG  GTGCTTTAGACGGTAGTTACCTGGT  TCCAAGTGATTTTCCATTTGTAGTA  GTGGACTTCGGAACAAGCTGA |
| CaSBP04 | SBPzunla-4-F  SBPzunla-4-R | ATGTTGGACTATGAATGGG  CTAATATGTTCTTTGCCTAAAAC |
| CaSBP07 | SBPzunla-7-F  SBPzunla-7-R | ATGGAAACAACTAATAACCAGC  TCAACGGTGGAAGTTTTTGT |
| CaSBP08 | SBPzunla-8-F  SBPzunla-8-R  SBPcm334-8-F  SBPcm334-8-R | ATGGCAACCCAAATCTATGG  TTAAATCGATACTCTACCACTGCTG ATGGACTTTGTTGAAGAAGACAATGAG  TCAAAAATAATGAGATGACATAGTTTTGTT |
| CaSBP09 | SBPcm334-9-F  SBPcm334-9-R | ATGAGAGGTGTTGGCAACAT  TCAGAGAGTGGAACTACAATATGC |
| CaSBP11 | SBPzunla-11-F  SBPzunla-11-R | TGGTTCAGTGGCAGGTGC  TCAAGTGATTCTAAGGCCGG |
| CaSBP12 | SBPzunla-12-F  SBPzunla-12-R | ATGTTGGACTATGACTGGGGAG  TTATGGTCTTTGCCTAAAAC |
| CaSBP13 | SBPzunla-13-F  SBPzunla-13-R | GGGCACTTGGAAGAAGTCTG  GTGTTGTCTTCCATCGCCAT |
| CaSBP15 | SBPcm334-15-F  SBPcm334-15-R | TCTGATGAGAAAACCAATGTTAGTGAC  GGACAGATTTCGGCACCCTC |
