# Supplementary material for: Genome-Wide Identification and Analysis of the SBP-Box Family Genes under Phytophthora capsici Stress in Pepper (Capsicum annuum L.)
Source: Front Plant Sci. 2016 Apr 15;7:504. doi: 10.3389/fpls.2016.00504 (PMC4832253; doi:10.3389/fpls.2016.00504)
Supplement: Supplementary file 2 [file Table_2.DOC]

**Table S2 Primers names and their sequences used in study for quantitative PCR analysis.**

| Oligo Name | Primer Abbreviation | Primer Sequence (5^’-^3’) |
| --- | --- | --- |
| CaSBP01 | RTSBPzunla-1-F  RTSBPzunla-1-R | AGTGGTGGTTATGGTTCTGATGTGA  CCAATGGTCTCACAGATCCGCT |
| CaSBP02 | RTSBPzunla-2-F  RTSBPzunla-2-R | CTCCAACGGAACAAACCCCAC  GAGGTGCCTGTGAACCATTTGAG |
| CaSBP03 | RTSBPzunla-3-F  RTSBPzunla-3-R | ATGTTGGATGTGGCGTGGTTAC  GATTTGACTAGAAGTATTTGCCTCC |
| CaSBP04 | RTSBPzunla-4-F  RTSBPzunla-4-R | ATGACAAATTATCAACACCAGCA  AAGCAAAATACGTTCTCCCG |
| CaSBP05 | RTSBPzunla-5-F  RTSBPzunla-5-R | ATGGACACTAACAAATGGGAAGG  GATGTGATGACCCTCCACCAGA |
| CaSBP06 | RTSBPzunla-6-F  RTSBPzunla-6-R | TTTGGCATACTGGATGGGTTTA  CCTTCCAGGGCACATAATAACC |
| CaSBP07 | RTSBPzunla-7-F  RTSBPzunla-7-R | AACAACTAATAACCAGCTGAAATCC  TTATGCCTCTTATGGTACTGCTTAG |
| CaSBP08 | RTSBPzunla-8-F  RTSBPzunla-8-R | ATGGCAACCCAAATCTATGGT  TATCACTCTTTTCCCCCTTTTTCTT |
| CaSBP09 | RTSBPzunla-9-F RTSBPzunla-9-R | TTGGTAGGTGTATTTTCTGGTG  ACAAGTAGTGCCTGCTGTATTC |
| CaSBP10 | RTSBPzunla-10-F  RTSBPzunla-10-R | GGACGACGAGGAAGACGAAGAT  TCGAACTCCGCCAACTGATGAA |
| CaSBP11 | RTSBPzunla-11-F  RTSBPzunla-11-R | AAGGACTGCCTGCCGTAACAAC  GACGAGCCCTGTGATTGAGATG |
| CaSBP12 | RTSBPzunla-12-F  RTSBPzunla-12-R | ATCCTCCGTTATGCTTTCTGGC  TACCTTGGGAATGGGTGAAACA |
| CaSBP13 | RTSBPzunla-13-F  RTSBPzunla-13-R | ATCTCTTTTCTCTACACACTATGGG  GAATCTGAGACTCCAGGAAAACA |
| CaSBP14 | RTSBPzunla-14-F  RTSBPzunla-14-R | CTATCATCGGGTTCTATGTTTACG  TGATTATTCTTGCCGTCCCTAT |
| CaSBP15 | RTSBPcm334-15-F  RTSBPcm334-15-R | CATTTTTGGTTCAGTGGTAGGT  AGACTGAATTCTGCCTTGGTA |
