# Supplementary material for: Genome-Wide Identification and Analysis of the SBP-Box Family Genes under Phytophthora capsici Stress in Pepper (Capsicum annuum L.)
Source: Front Plant Sci. 2016 Apr 15;7:504. doi: 10.3389/fpls.2016.00504 (PMC4832253; doi:10.3389/fpls.2016.00504)
Supplement: Supplementary file 3 [file Table_3.DOC]

**Table S3** General information of SBP-box genes selected for phylogenetic analysis.

| Gene | Accession no. or locus ID | Protein sequence of SBP-domain |
| --- | --- | --- |
| CaSBP01 | Capana01g002647 | CQVEGCNLVLSSAKEYYRKHRVCDSHSKCPKVIVAGVARRFCQQC  SRFHSVSEFDEKKRSCRRRLSDHNARRRKPQQE |
| CaSBP02 | Capana01g002832 | CQVDDCGTDLSKAKDYHRRHKVCEMHSKASRALVGNVMQRFCQQC  SRFHALQEFDEGKRSCRRRLAGHNKRRRKTQSE |
| CaSBP03 | Capana01g003073 | CQVPDCEADISELKGYHKRHRVCLRCANASAVLLDGHNKRYCQQC  GKFHILSDFDEGKRSCRRKLERHNNRRRRKATD |
| CaSBP04 | Capana01g003445 | CQAEGCNADLTHAKHYHRRHKVCEFHSKAATVVAAGLTQRFCQQC  SRFHVLTEFDNGKRSCRKRLADHNRRRRK |
| CaSBP05 | Capana02g001917 | CQVEQCTADMANAKPYHRRHKVCEFHSKSSIVLISGLQQRFCQQC  AEFDEAKRSCRRRLAGHNERRRKISYD |
| CaSBP06 | Capana05g002237 | CQVQDCQVDLSMSKDYHRRHKVCGVHSKAAQALVGNVMQRFCQQC  SRFHVLEEFDEGKRSCRRRLAGHNKRRRKTNPE |
| CaSBP07 | Capana07g001731 | CQAEKCSVDLNDAKQYHKRHKVCENHAKSQVVVVAGLRQRFCQQC  SRFHELTEFDESKRSCRRRLAGHNERRRKS |
| CaSBP08 | CA07g17550 | CQVEKCGVDLDGAKKYYKRHKVCQLHAKAPIVLLAGLRHRFCQQC  SRY |
| CaSBP09 | CA08g03640 | RFHLLDEFDDGKQSYRKHLVGHNEHQRKPH |
| CaSBP10 | Capana10g000507 | CQVEDCTADMVDAKAYHRRHKVCEFHAKAPAVPIGGLLQRFCQQC  SRFHQLAEFDDTKRSCRRRLAGHNERRRKSAHD |
| CaSBP11 | Capana10g000709 | CQVQGCGKDLSPCKDYHKRHKVCEVHSKTAKVIVNGIEQRFCQQC  SRFHLLAEFDDGKRSCRKRLAGHNERRRKPH |
| CaSBP12 | Capana10g000886 | CQVEGCSADLTHAKHYHRRHKVCEFHSKASTVIAAGLTQRFCQQC  SRFHLLSEFDNGKRSCRKRLADHNRRRRKNQQA |
| CaSBP13 | Capana10g002379 | CQVEGCQADLSDAKAYYSRHKVCGMHSKSPTAVVAGLEQRFCQQC  SRFHQLPEFDQGKRSCRRRLACHNERRRKPP |
| CaSBP14 | Capana11g002003 | CQVHGCNKDLSSSKDYHKRHKVCDEHSKTAIVIVNGIEQRFCQQC  SRFHLLAEFDEGKRSCRKRLAGHNERRRKPQFD |
| CaSBP15 | CA11g04690 | CQVEQCTADMANAKPYHRRHKVCEFHSKSSIVLISGLQQRFCQQC  SRFHLLAEFDEAKRSCRRRLAGHNERRRKISYD |
| OsSPL1 | LOC_Os01g18850.1 | CQVDGCTVNLSSARDYNKRHKVCEVHTKSGVVRIKNVEHRFCQQC  SRFHFLQEFDEGKKSCRSRLAQHNRRRRKVQ |
| OsSPL2 | LOC_Os01g69830.1 | CSVEGCAADLSKCRDYHRRHKVCEAHSKTAVVTVAGQQQRFCQQC  SRFHLLGEFDEEKRSCRKRLDGHNKRRRKPQ |
| OsSPL3 | LOC_Os02g04680.1 | CQVEGCNVDLSSAKPYHRKHRVCEPHSKTLKVIVAGLERRFCQQC  SRFHGLAEFDQKKRSCRRRLHDHNARRRKPQ |
| OsSPL4 | LOC_Os02g07780.1 | CQVEGCGVELVGVKDYHRKHRVCEAHSKFPRVVVAGQERRFCQQC  SRFHALSEFDQKKRSCRRRLYDHNARRRKPQ |
| OsSPL5 | LOC_Os02g08070.1 | CQAEGCKADLSAAKHYHRRHKVCDFHAKAAAVLAAGKQQRFCQQC  SRFHVLAEFDEAKRSCRKRLTEHNRRRRKPT |
| OsSPL6 | LOC_Os03g61760.1 | CQVEGCTADLTGVRDYHRRHKVCEMHAKATTAVVGNTVQRFCQQC  SRFHPLQEFDEGKRSCRRRLAGHNRRRRKTR |
| OsSPL7 | LOC_Os04g46580.1 | CQVEGCDITLQGVKEYHRRHKVCEVHAKAPRVVVHGTEQRFCQQC  SRFHVLAEFDDAKKSCRRRLAGHNERRRRSN |
| OsSPL8 | LOC_Os04g56170.1 | CQAEGCKADLSSAKRYHRRHKVCEHHSKAPVVVTAGLHQRFCQQC  SRFHLLDEFDDAKKSCRKRLADHNRRRRKSK |
| OsSPL9 | LOC_Os05g33810.1 | CQVPGCEADIRELKGYHRRHRVCLRCAHAAAVMLDGVQKRYCQQC  GKFHILLDFDEDKRSCRRKLERHNRRRRKPD |
| OsSPL10 | LOC_Os06g44860.1 | CQAEGCKADLSGAKHYHRRHKVCEYHAKASVVAASGKQQRFCQQC  SRFHVLTEFDEAKRSCRKRLAEHNRRRRKPA |
| OsSPL11 | LOC_O06g45310.1 | CQVEGCGLELGGYKEYYRKHRVCEPHTKCLRVVVAGQDRRFCQQC  SRFHAPSEFDQEKRSCRRRLSDHNARRRKPQ |
| OsSPL12 | LOC_Os06g49010.1 | CQVEGCKVDLSSAREYHRKHKVCEAHSKAPKVIVSGLERRFCQQCS  RFHGLAEFDQKKKSCRRRLSDHNARRRKPQ |
| OsSPL13 | LOC_Os07g32170.1 | CQVERCGVDLSEAGRYNRRHKVCQTHSKEPVVLVAGLRQRFCQQC  SRFHELTEFDDAKRSCRRRLAGHNERRRKSA |
| OsSPL14 | LOC_Os08g39890.1 | CQVEGCGADLSGIKNYYCRHKVCFMHSKAPRVVVAGLEQRFCQQC  SRFHLLPEFDQGKRSCRRRLAGHNERRRRPQ |
| OsSPL15 | LOC_Os08g40260.1 | CQVDDCRADLTNAKDYHRRHKVCEIHGKTTKALVGNQMQRFCQQC  SRFHPLSEFDEGKRSCRRRLAGHNRRRRKTQ |
| OsSPL16 | LOC_Os08g41940.1 | CAVDGCKEDLSKCRDYHRRHKVCEAHSKTPLVVVSGREMRFCQQC  SRFHLLQEFDEAKRSCRKRLDGHNRRRRKPQ |
| OsSPL17 | LOC_Os09g31438.1 | CQVEGCGVDLSGVKPYYCRHKVCYMHAKEPIVVVAGLEQRFCQQC  SRFHQLPEFDQEKKSCRRRLAGHNERRRKPT |
| OsSPL18 | LOC_Os09g32944.1 | CAVDGCKADLSKHRDYHRRHKVCEPHSKTPVVVVSGREMRFCQQC  SRFHLLGEFDEAKRSCRKRLDGHNRRRRKPQ |
| OsSPL19 | LOC_Os11g30370.1 | CSVDGCRSDLSRCRDYHRRHKVCEAHAKTPVVVVAGQEQRFCQQC  SRFHNLAEFDDGKKSCRKRLDGHNRRRRKPQ |
| Solyc07g062980 | Solyc07g062980 | CQVETCEANLDGAKKYHKRHKVCQVHAKAPIVLLAGLKQRFCQQC  SKFHELSEFDGTKKSCRLRLDGHNKRRRKTP |
| Solyc04g064470 | Solyc04g064470 | HVLEEFDEGKRSCRRRLTGHKKRRRKTHPE |
| CNR | Solyc02g077920 | CQVDQCTADMADAKPYHRRHKVCEFHSKSPIVLISGLQKRFCQQC  SRFHLLAEFDDAKRSCRRRLAGHNERRRKIT |
| SlySBP2 | Solyc04g045560 | CQVDSCNLDLSSAKQYHRKHRVCHIHSKCPKVIILGRHRRFCQQC  SRFHSLSDFDENKRSCRTRLSDHNARRRKPQ |
| SlySBP3 | Solyc10g009080 | CQVEECTADMVNAKTYHRRHKVCEFHAKAPEVLIDGLRQRFCQQC  SRFHQLAEFDDAKKSCRRRLAGHNERRRKSA |
| SlySBP4 | Solyc07g053810 | CQAEKCNVDLSDAKQYHKRHKVCEYHAKSQVVVVAGLRQRFCQQC  SRFHELTEFDESKRSCRRRLAGHNERRRKST |
| SlySBP6a | Solyc03g114850 | CQVQGCGKDLSPCKDYHKRHKVCEVHSKTAKVIVNGIEQRFCQQC  SRFHLLAEFDDGKRSCRKRLAGHNERRRKPP |
| SlySBP6b | Solyc05g012040 | CQVHGCNKDLSSSKDYHKRHKVCNEHSKTAIVIVNGIEQRFCQQC  SRFHLLAEFDEGKRSCRKRLAGHNERRRKPQ |
| SlySBP6c | Solyc12g038520 | CQVQGCGKDLTSCKDYHKRHKVCEIHSKTAKVIVNGIQQRFCQQC  SRFHLLAEFDDGKRSCRKRLAGHNERRRKPH |
| SlySBP7 | Solyc01g080670 | CQVPDCEADISELKGYHKRHRVCLRCANATSVVLDGHSKRYCQQC  GKFHILSDFDEGKRSCRRKLERHNNRRRRKA |
| SlySBP8a | Solyc10g018780 | CQAEGCNADLTHAKHYHRRHKVCEFHSKASTVIAAGITQRFCQQC  SRFHLLSEFDNGKRSCRKRLADHNRRRRKNQ |
| SlySBP8b | Solyc01g090730 | CQAEGCNADLTHAKHYHRRHKVCEFHSKASTVIAAGLTQRFCQQC  SRFHVLSEFDNGKRSCRKRLADHNRRRRKNI |
| SlySBP10 | Solyc05g015510 | CQVEGCNLDLSSAKEYYRKHRVCDSHSKSPKVIVAGVARRFCQQC  SRFHSVSEFDDKKRSCRRRLSDHNARRRKPH |
| SlySBP12a | Solyc01g068100 | CQVDDCGTDLSKAKDYHRRHKVCEMHSKASRALVGNVMQRFCQQC  SRFHALQEFDEGKRSCRRRLAGHNKRRRKTQ |
| SlySBP12b | Solyc05g053240 | CQVQDCRADLSSAKDYHRRHKVCEVHSKAAKALVGNVMQRFCQQC  SRFHVLEEFDEGKRSCRRRLAGHNKRRRKTH |
| SlySBP13 | Solyc05g015840 | CLVDGCNADLSECREYHRRHKVCEVHSKTAKVTIAGRDQRFCQQC  SRFHSLVEFDDGKRSCRKRLDGHNRRRRKPQ |
| SlySBP15 | Solyc10g078700 | CQVEGCQADLSDAKAYYSRHKVCGMHSKSPTVVVAGLEQRFCQQC  SRFHQLTEFDQGKRSCRRRLACHNERRRKPP |
| AtSPL1 | At2g47070 | CQVENCEADLSKVKDYHRRHKVCEMHSKATSATVGGILQRFCQQC  SRFHLLQEFDEGKRSCRRRLAGHNKRRRKTN |
| AtSPL2 | At5g43270 | CQVEGCNLDLSSAKDYHRKHRICENHSKFPKVVVSGVERRFCQQC  SRFHCLSEFDEKKRSCRRRLSDHNARRRKPN |
| AtSPL3 | At2g33810 | CQVESCTADMSKAKQYHKRHKVCQFHAKAPHVRISGLHQRFCQQC  SRFHALSEFDEAKRSCRRRLAGHNERRRKST |
| AtSPL4 | At1g53160 | CQVDRCTADMKEAKLYHRRHKVCEVHAKASSVFLSGLNQRFCQQC  SRFHDLQEFDEAKRSCRRRLAGHNERRRKSS |
| AtSPL5 | At3g15270 | CQVDRCTVNLTEAKQYYRRHRVCEVHAKASAATVAGVRQRFCQQC  SRFHELPEFDEAKRSCRRRLAGHNERRRKIS |
| AtSPL6 | At1g69170 | CQVYGCSKDLSSSKDYHKRHRVCEAHSKTSVVIVNGLEQRFCQQC  SRFHFLSEFDDGKRSCRRRLAGHNERRRKPA |
| AtSPL7 | At5g18830 | CQVPDCEADISELKGYHKRHRVCLRCATASFVVLDGENKRYCQQC  GKFHLLPDFDEGKRSCRRKLERHNNRRKRKP |
| AtSPL8 | At1g02065 | CQAEGCNADLSHAKHYHRRHKVCEFHSKASTVVAAGLSQRFCQQC  SRFHLLSEFDNGKRSCRKRLADHNRRRRKCH |
| AtSPL9 | At2g42200 | CQVEGCGMDLTNAKGYYSRHRVCGVHSKTPKVTVAGIEQRFCQQC  SRFHQLPEFDLEKRSCRRRLAGHNERRRKPQ |
| AtSPL10 | At1g27370 | CQIDGCELDLSSSKDYHRKHRVCETHSKCPKVVVSGLERRFCQQC  SRFHAVSEFDEKKRSCRKRLSHHNARRRKPQ |
| AtSPL11 | At1g27360 | CQIDGCELDLSSAKGYHRKHKVCEKHSKCPKVSVSGLERRFCQQC  SRFHAVSEFDEKKRSCRKRLSHHNARRRKPQ |
| AtSPL12 | At3g60030 | CQVDNCGADLSKVKDYHRRHKVCEIHSKATTALVGGIMQRFCQQC  SRFHVLEEFDEGKRSCRRRLAGHNKRRRKAN |
| AtSPL13A | At5g50570 | CLVDGCDSDFSNCREYHKRHKVCDVHSKTPVVTINGHKQRFCQQC  SRFHALEEFDEGKRSCRKRLDGHNRRRRKPQ |
| AtSPL13B | At5g50670 | CLVDGCDSDFSNCREYHKRHKVCDVHSKTPVVTINGHKQRFCQQC  SRFHALEEFDEGKRSCRKRLDGHNRRRRKPQ |
| AtSPL14 | At1g20980 | CQVDNCTEDLSHAKDYHRRHKVCEVHSKATKALVGKQMQRFCQQC  SRFHLLSEFDEGKRSCRRRLAGHNRRRRKTT |
| AtSPL15 | At3g57920 | CQVEGCRMDLSNVKAYYSRHKVCCIHSKSSKVIVSGLHQRFCQQC  SRFHQLSEFDLEKRSCRRRLACHNERRRKPQ |
| AtSPL16 | At1g76580 | CQVDNCKEDLSIAKDYHRRHKVCEVHSKATKALVGKQMQRFCQQC  SRFHLLSEFDEGKRSCRRRLDGHNRRRRKTQPD |
